# Editorial: Nanomedicine in Infectious Diseases: Drug Delivery and Vaccines

**DOI:** 10.3389/fphar.2022.928572

**Published:** 2022-08-17

**Authors:** Srujan Marepally, Tejram Sahu, Rajeev K. Tyagi

**Affiliations:** ^1^ Centre for Stem Cell Research (CSCR) (a Unit of InStem, Bengaluru), Vellore, India; ^2^ Department of Molecular Microbiology and Immunology, Johns Hopkins Malaria Research Institute, Johns Hopkins University Bloomberg School of Public Health, Baltimore, MD, United States; ^3^ Biomedical Parasitology and Nano-immunology Lab, Division of Cell Biology and Immunology, CSIR-Institute of Microbial Technology (IMTECH), Chandigarh, India

**Keywords:** nanoscale drug carriers, inflammation, pseudovirus, tuberculosis, gene editing, anti-bacterial therapy

## Introduction

The Research Topic “Nanomedicine in infectious diseases: Drug delivery and vaccines” focuses on the role of nano-formulations in the delivery of candidate vaccines and drugs to develop interventional approaches against infectious diseases. It includes eight original and review articles.

Infectious diseases such as Tuberculosis (TB), a contagious disease caused by the *Mycobacterium tuberculosis* (Mtb), are one of the major reasons for increased mortality in developing nations. The delivery of drugs to the site of disease is a challenge to achieving its therapeutic effects. Hence, efforts have been made to augment drugs and make them available at the disease site using lipid-based nanoscale drug delivery systems (NDDS). Nanocarrier-based therapeutics help overcome the toxicity and poor solubility issues of several drugs used for developing therapeutic interventions against tuberculosis (Rajput et al.). Diverse nanoscale carriers and their use in drug and vaccine delivery and how they have evolved to overcome the challenges associated with sustained and target specific delivery, stability, durability, efficacy, and bio-distribution. They also enable drugs to be taken up by the active macrophages (Rajput et al.), which are used as target sites for the active and passive targeting of nanocarriers. Nanocarriers are anchored with the target specific ligand for the sustained and target specific delivery of a drug(s) and antigens for effective delivery (Limocon et al.). These ligand anchored nanocarriers are made up of chitosan and enhance the drug concentration locally and systemically and this delivery system mediated drug delivery increases the therapeutic potential for treating TB (Limocon et al.).

Aceclofenac (ACE), a cyclooxygenase-2 inhibitor, is a derivative of the diclofenac group that has been used for the symptomatic treatment of systemic inflammatory autoimmune disease, rheumatoid arthritis (RA). Partial solubility, high lipophilic nature, and stability issues pose challenges for developing topical formulations. Therefore, Garg et al. developed and characterized the nanostructured lipid carrier (NLC)–based ACE (ACE-NLC) hydrogel for their efficient transdermal delivery. NLC microemulsion was prepared using different lipids by various methods and was characterized with respect to particle size, zeta potential, surface morphology, and drug encapsulation efficiency (Garg et al.). The optimized NLC formulation was incorporated into Carbopol^®^ 940 gel, and this arrangement was characterized and compared with the existing marketed gel (Mkt-gel) formulation. The *in vitro*, *ex-vivo* dermato-kinetic modeling and *in vivo* skin retention, permeation, and stability confirmed the value of the NLC formulation loaded with aceclofenac for better skin distribution in the epidermis and dermis. The results of these findings showed that ACE-NLC permeated deeper into the skin layers and kept the skin integrity intact. Overall, the NLC-based gel formulation of ACE might be a promising nanoscale lipid carrier for topical application compared to the conventional Mkt-gel formulation (Garg et al.).

The nanocarriers mediated delivery of vaccine formulations through oral, nasal, and aerosol routes of administration was able to elicit antigen specific immune responses (Tyagi et al., 2015; Chaudhari et al., 2021). Nanocarriers improve the bioavailability and serve as adjuvants to elicit long-lasting and neutralizing immune responses, increasing the effectiveness of vaccines (Tyagi et al., 2015; Chaudhari et al., 2021). Drugs and vaccines with lower penetration abilities may also be delivered transmucosally while maintaining their biological functions. The most recent investigations on needle-free and non-invasive approaches to the delivery of vaccines using an oral trans-mucosal route, their strengths, and associated challenges is another upcoming field discussed in the special issue (Mangla et al.). Oral transmucosal vaccine delivery by nanocarriers is one of the most promising advancements in the field of vaccine delivery (Mangla et al.) and will help to decide future potential drug and vaccine trials in the field.

## Aerosolizable Lipid-Nanovesicles for Drug Delivery

The entire world has recently been witnessing an unprecedented upsurge in microbial lung infections. The major challenge encountered in treating the same is to ensure the optimum drug availability at the site of infection. Kaur et al. have effectively shown that aerosolization of antimicrobials has shown immense potential owing to their localized and targeted effect (Kaur et al.). Hence, efforts have been made to systematically develop lung-phosphatidylcholine-based lipid nanovesicles (NLVs) of voriconazole for potential management of super-infections like aspergillosis (Kaur et al.). Voriconazole loaded LNVs prepared by the thin-film hydration method exhibited improved *in vitro* antifungal activity. Safety and uptake studies on airway-epithelial cells indicate LNVs immense potential to permeate the cellular barrier of the lungs and pharmacokinetic studies have shown marked improvements in the retention profile of voriconazole in the lungs following LNVs nebulization compared to pristine voriconazole. Overall, LNVs have proven to be safe and effective delivery systems with the distinct potential to efficiently target respiratory fungal infections. This investigation confirms the value of voriconazole loaded lipid nanovesicles in treating lung infections.

## Polydopamine Nanocarriers for Antibacterial Drug Delivery

Polydopamine (PDA), a polymer of dopamine, is increasingly explored as a potential nanocarrier system for photodynamic therapy (PDT) and photothermal therapy (PTT) in anti-cancer and anti-bacterial applications. These polymeric nanocarrier systems showed high biocompatibility and their surface could be functionalized. Shuhao et al., summarize the various methods of fabrication of PDA nanosystems, their biological functions, and therapeutic applications, emphasizing antibacterial applications in their review (Fan et al.). Increasing bacterial resistance to the existing antibiotic agents is one of the major challenges faced by clinics. Promising pre-clinical data with PDA nanosystem-mediated PTT against intracellular bacteria may provide potential alternative therapies. However, extensive research on safety profiles is still warranted to translate PDA nanosystems into clinics (Fan et al.).

## Delivery of Gene Editing Reagents for Treating Viral Infections

Applications of CRISPR/Cas9 mediated gene editing are rapidly evolving for a number of treating genetic diseases and viral infections. Recent findings have successfully demonstrated the CRISPR/Cas9 system as an emerging novel antiviral approach for treating multiple infectious diseases such as severe acute respiratory syndrome coronavirus 2 (SARS-CoV2), human immunodeficiency virus (HIV), human papillomavirus (HPV), and many more as cleaving the active part of viral genome with molecular scissors leads to the inactivation of the virus.

Jinfeng et al. have similarly demonstrated an intriguing approach to treating cervical cancer and inactivating human papillomavirus (HPV) by delivering CRISPR/Cas9 reagents in plasmid DNA form through beta-amino ester polymeric nanoparticles (Xiong et al.). Their study convincingly demonstrated that the inhibition of tumor growth by restoration of p53 expression and HPV16 inactivation by inhibiting E7 protein, which plays a very critical role in viral replication using the CRISPR/Cas9 system, reduced cervical cancer progression significantly in a mouse model (Xiong et al.).

## SARS-CoV2 pseudovirus as a Tool to Evaluate COVID19 Therapeutics

SARS-CoV2 pseudoviruses are an important tool in assessing the efficacy of therapeutics and vaccines for COVID19. When developing a robust protocol to enable pseudovirus production for high-throughput screening, Mahalingam *et al.*, optimized a comprehensive protocol for SARS-CoV2 pseudovirus production in the lentivirus backbone that includes the development of a transfection reagent to transfect lentiviral plasmids and identifying best lentiviral plasmids for high titer virus production (Mahalingam et al.). The novel liposomal transfection reagent was found to be as efficient as the commercial transfection reagent, lipofectamine 3,000. An optimal lentivirus system containing spike protein expressing plasmids was used for high titer SARS-CoV2 pseudovirus production. The pseudovirus was validated with human sera. In conclusion, optimized protocol may be further explored in the high throughput screening of COVID19 therapeutics (Mahalingam et al.).

## Conclusion

This Research Topic collates data from manuscripts focusing on the use of nanoscale drug carriers as delivery systems (DS) for vaccines and drugs, which can be used for developing interventional approaches against infectious and inflammatory diseases. The importance of the transdermal route of administration for the delivery of anti-inflammatory drugs such as aceclofenac and cox-2 inhibitor, have been highlighted. This topic also covers antibacterial (tuberculosis) and antiviral drug delivery, particularly the SARS-CoV2 pseudovirus, as a tool for assessing COVID19 therapeutics. Overall, the role of nanomedicine in the treatment of infectious and inflammatory diseases should be explored further in the near future.
